# Pharmaceutical regulation in Europe and its impact on corporate R&D

**DOI:** 10.1186/s13561-014-0023-5

**Published:** 2014-10-03

**Authors:** Stephan Eger, Jörg C Mahlich

**Affiliations:** Medical University of Vienna, Spitalgasse 23, Vienna, 1090 Austria; University of Vienna, Department of Economics, Oskar-Morgenstern-Platz 1, Vienna, 1090 Austria

**Keywords:** Pharmaceutical Industry, R&D investments, Regulation

## Abstract

**Objectives:**

Many European countries regulate the markets for prescription drugs in order to cope with rising health expenditures. On the other hand, regulation distorts incentives to invest in pharmaceutical R&D. This study aims at empirically assessing the impact of regulation on pharmaceutical R&D expenditures.

**Methods:**

We analyze a sample of 20 leading pharmaceutical companies between 2000 and 2008. The share of sales in Europe serves as a proxy for the degree of pharmaceutical regulation. We control for other firm specific determinants of R&D such as cash flow, company size, leverage ratio, growth rate, and Tobin’s q.

**Results:**

Our results suggest a nonlinear relationship between European sales ratio and R&D intensity. Beyond a threshold of 33% of sales generated in Europe, a higher presence in Europe is associated with lower R&D investments.

**Conclusion:**

The results can be interpreted as further evidence of the deteriorating effect of regulation on firm’s incentives to invest in R&D.

## Background

In order to reduce pressure on public health care expenditures many European countries regulate their markets for pharmaceuticals. Both theoretical and empirical studies show that this approach is indeed associated with lower drug prices and consecutively with lower health expenditures. Wright [1] for instance applies a game theoretic framework for modeling price negotiations between a pharmaceutical company and the regulator. He finds that in equilibrium regulation leads to lower prices compared to unregulated markets. This theoretical finding is empirically backed by Danzon and Chao [2] who demonstrate that countries with strict regulation such as France or Italy exhibit lower drug prices than the less regulated market of the United States. Lower prices in turn make it more difficult for firms to redeem the rising research and development (R&D) costs. It is estimated that on average expenditures of 1 billion U.S. dollars are needed to bring a new molecule to the market [3,4]. A regulatory regime that leads to lower drug prices can distort incentives to invest in R&D, which might incur long run economic costs induced by a future absence of new drugs and consecutive lost life years [5]. In this paper we try to quantify the effect of pharmaceutical regulation in Europe on corporate R&D investments. We follow the approach of Vernon [6,7] and take the share of companies’ sales made in Europe as a proxy for the average degree of European drug regulation. The firm’s share of sales in enlarged Europe is supposed to impact its R&D intensity directly. Hence, the higher the share of sales a firm generates in Europe, the lower is its overall profitability, and the lower is its R&D intensity. This relationship has not been studied directly and is the primary differentiation to the work of Vernon. Vernon (2003) examined the impact of non-US sales on profits [6] while Vernon (2005) simulated a drop in pharmaceutical profit margins of U.S. firms to the level of profit margins in markets outside the U.S. He then estimated the consequences with regard to pharmaceutical R&D [7].

Our analysis only aims at assessing the relationship between regulation and corporate R&D. We do not examine social welfare effects. In the framework of welfare economics R&D investments constitute costs which can be balanced out by potential benefits of future drug innovations. However, due to long time horizons and high uncertainty statements on social welfare effects are difficult and for this reason disregarded in this analysis. The remainder of the paper proceeds as follows. We briefly describe pharmaceutical regulation in European countries and review the literature on the impact of regulation and R&D investments. Then, we introduce our empirical approach and present the results. The paper closes with a discussion of our findings.

### Regulatory framework in Europe

Pharmaceutical price regulation in Europe has attracted attention both from academics and policy makers [8-13]. Most European countries employ a huge variety of regulation measures at the same time both on the demand and on the supply side. Table 1 provides an overview of different regulation mechanisms that were in place in Europe as of 2010. In the meantime, Germany has introduced a mandatory Health-Technology Assessment (HTA) as well. Known as the ‘Arzneimittelmarktneuordungsgesetz’ (AMNOG) or ‘Pharmaceutical Market Reorganization Act’ the new regulation has introduced an early benefit assessment of new drugs and links prices to the degree of added benefit against a comparator drug [14,15]. So far, in only 40% of the assessments an additional benefit was granted [16]. While the main motivation of the new law was to save up to 2 billion Euro annually for the statutory health insurance due to heavy deficits at that time [17,18], empirical evidence is scarce whether this goal has been achieved. Only Aggerwal [19] claimed to observe a decline of German drug prices due to AMNOG. As Germany is a major reference country for other pharmaceutical markets, this would have a considerable impact on international prices as well. As Stargardt and Schreyögg showed, for every Euro the price is lowered in Germany, the reimbursement price in other countries decreases up to 36 Eurocent [20].

**Table 1:** **Overview of pharmaceutical regulation in Europe**

**Instrument**	**Countries**
Supply side regulation: in patent drugs	
Price controls: administrative or statutory pricing	All EU Member States except Germany, UK and, to a certain extent, Sweden
External reference pricing	All EU Member States except UK, Germany, Sweden
Rate of return regulation	UK
Negotiations and price-volume agreements	France, Italy, Austria
Direct expenditure controls: payback	France, Portugal, Austria
Direct expenditure controls: price volume agreements	France
Cost-plus pricing	Spain
Supply side regulation: off patent drugs	
Tendering for generics pharmaceuticals in primary care	Netherlands, Germany
Price capping for generics and linking these to the originator price	Italy, Greece, France
Supply side regulation: reimbursement methods	
Positive and negative formularies	All EU Member States
Internal reference pricing	Germany, Netherlands, Czech Republic, Italy, Spain, France, Hungary
Health Technology Assessments (HTA)	UK, Sweden, Netherlands, Hungary, Poland, Finland, Estonia, Latvia, Lithuania. In France only assessment of clinical benefit
Innovative pricing and reimbursement schemes	Italy, Germany, UK, Finland
Demand side regulation: policies towards physicians	
Clinical practice guidelines	All EU Member States
Compulsory generic prescribing	UK, Denmark, Estonia
Financial incentives	France, UK
Prescription monitoring and audit	Belgium, UK, Netherlands, France, Denmark, Sweden, Estonia
Demand side regulation: policies towards pharmacies	
Control of remuneration (e.g. margins, fees) including contractual arrangements	All Member States
Generic substitution	France, Italy, Spain, Sweden
Demand side regulation: policies towards patients	
Cost-sharing	All EU Member States
Encouraging use of over-the counter medicines and “de-listing”	UK, Germany, Sweden, Netherlands

Source: Kanavos et al. [11].

As both the supply and demand side of the market is strongly regulated it is difficult to evaluate the effect of a specific regulatory action. For this reason, our approach is to take the set of regulatory measures as a whole and compare it with the average degree of regulation in other regions of the world. European regulation is successful in the sense that it does lower prices compared to an unregulated environment. Price comparisons show that retail prices for branded prescription medicines in the United States are higher than those in key European markets [21-23]. Comparing wholesale drug prices in 9 European countries Martikanen et al. [24] confirm that prices were highest in those countries where manufacturers are relatively free to set the prices of their products. At the time of their study this was the case in the UK, Sweden and Denmark. The study of Schulenburg et al. [25] suggests that supply side measures are more effective than demand side measures in reducing pharmaceutical prices. Sood et al. [26] were able to show that different regulative measures have different effects on pharmaceutical revenues with direct price controls having the largest negative impact, followed by economic evaluations and budgets.

### Impact on R&D

Pharmaceutical regulation is associated with lower drug prices. This does not necessarily lead to a reduction of pharmaceutical sales as standard economic theory suggests that lower prices increase demand in quantity units. Hart et al. [27] for instance found that the price of a treatment does influence physician’s prescription behavior. In a framework of a meta-analysis Marin et al. [28] confirm a negative price elasticity of pharmaceuticals. Their estimates suggest that a price increase of 1% would reduce demand by 0.209%. Accordingly, an empirical study by Stremersch and Lemmens [29] found manufacturer price controls to have a small albeit positive effect on drug sales measured in quantity units. However, it seems that the additional demand which is triggered by lower prices does not compensate the price effect and regulation exerts an overall negative influence on cash flows and subsequently reduces corporate profit margins [6]. A reduction of profit margins in turn has a direct impact on corporate R&D investment decisions. Theoretical work on this topic has been introduced for instance by Filson and Masia [30] who present a computational model in which even small reductions in profitability have substantial impacts on firm success and innovation. By means of a Markov model Filson [31] simulates what happened if the U.S. adopted price controls like those in the rest of the world. According to his calculation, this measure would reduce the number of new drugs by approximately 75%.

A number of empirical papers confirm the positive relation between profitability and R&D investments. For U.S. pharmaceutical firms Scherer [32] reports a simple Pearsonian correlation between gross profitability and R&D outlays of +0.92. Trushin [33] and Scherer [34] find that a 10% cash flow increase gives rise to an increase of R&D investments in the range of 3.6% and 6.1%. A similar value of 5.8% is derived for US medical device companies [35]. From an industry perspective, a 10% increase of the profit margin leads to a 7.7% boost of R&D investments in the US [36]. A similar relationship has been confirmed for the Japanese pharmaceutical industry [37]. This relates to the finding of Acemoglu and Linn [38] who also show that greater profitability spurs faster innovation. In their study a 1 percent increase in the potential market size for a drug category leads to a 4 to 7.5 percent increase in the number of new drugs in that category.

Some studies directly estimate the link between prices and R&D investments. According to Giaccotto et al. [39] a 10% drug price increase corresponds with a 5.8% increase of R&D expenditures. Another interesting study in this context is from Civan and Maloney [40]. Drawing on a cross sectional analysis of the prices of 600 drugs they tried to estimate the relation between the price level of already marketed drugs and the number of new pipeline drugs under development. A 10% price decrease goes along with a 2.8% to 4.9% decline of pipeline drugs.

When it comes to a comparison between Europe and the U.S. Vernon’s [6] results suggest that an increase of 10% in share of sales made in the non U.S. market result in a decline of 2.7% to 3.5% in profit margins. He used a panel data set containing the 20 biggest pharmaceutical companies from 1994 to 1999. The main assumption was that drug prices in the U.S. largely remain unregulated compared with the rest of the world, therefore he used the share of companies’ sales made in the non U.S. market as indicator of regulation. In a next step Vernon [7] identifies lagged cash flow and expected profits to be key determinants of pharmaceutical companies’ R&D spending. Both R&D drivers identified are influenced by regulation. For his estimation he used a panel data set of the 14 biggest pharmaceutical companies in the period of 1994 to 1997. Again, he assumes drug prices in the U.S. to be less regulated compared to the rest of the world. He then tried to simulate how a new policy regulating pharmaceutical prices in the U.S. affects R&D investment. According to this simulation, a policy which would regulate the U.S. prices in a way equivalent to the rest of the world would results in a decline in firms’ R&D expenditures in the range of 23.4 and 32.7 percent. Golec and Vernon [41] estimate the forgone R&D investments in Europe that have been induced by European price regulation. Between 1986 und 2004 European price regulation has impeded $4.96 bn (in 1986 $) of R&D investments according to their simulations. Those forgone investments would have resulted in 49 new drugs. A similar study was introduced by Brouwers et al. [42] who state that the drug price level within the OECD countries would have been 35%-45% higher in the absence of price regulation. Higher prices would have triggered additional annual R&D investments of $17–22 bn which in turn would have resulted in ten to thirteen new drug introductions per year. Another exciting work was published by Abbot and Vernon [5]. They perform a prospective micro-simulation and use Monte Carlo simulation techniques. According to their results, a price cut of 40 to 50 percent of drug prices in the U.S. would lead to a decline between 30 and 60 percent of investments in early-stage development projects.

Yet another approach to capture the impact of price regulation on R&D spending was introduced by Santerre et al. [43]. The authors argue that even in the U.S. market a certain kind of government influenced is asserted on drug prices. Bargaining power of the national Centers of Medicaid and Medicare services result in lower drug prices. According to their estimations drug prices would be 28% higher in the absence of Medicare/Medicaid programs. Due to the lower drug prices $256 bn of R&D investments have been withheld.

Summing up, the narrative review of the existing literature reveals detrimental effects of price regulation on R&D investments. This finding leads us to formulate the following hypothesis:

**Hypothesis:***Due to pharmaceutical regulation in Europe we expect that firms which make a bigger fraction of their sales in Europe do invest less in R&D as they are not able to fully recoup their R&D expenditures.*

We do expect that this relationship does not exhibit a linear relationship because R&D costs can be regarded as sunk costs and globally operating firms need to sell their products in all major markets as long as the marginal net returns exceed the marginal costs of selling. The a priori incentives to invest in R&D, on the other hand, are greater for firms whose products fit the specific medical needs of the U.S. patients and therefore are overrepresented in the U.S. market.

## Methods

We analyze a panel data set that contains annual information of a sample of the world’s biggest 20 pharmaceutical companies between 2000 and 2008, namely Abbot, Amgen, Astellas, AstraZeneca, Baxter, Bayer, BristolMyersSquibb, Eisai, Eli Lilly, GlaxoSmithKline, Johnson & Johnson, Merck, Novartis, Novo Nordisk, Pfizer, Roche, Sanofi-Aventis, Schering-Plough, Takeda, and Wyeth. All financial data are from the Compustat Global database. Regional market share data are from IMS, an international private market research company. The observation time period was selected because of data availability and because the absence of merger activities that would have complicated our analysis.

In line with Vernon [6,7], we take the share of companies’ sales made in Europe as an indicator of average European regulation. To test the relationship between European sales and R&D investments we put equation (1) forward:

1 $$ R{D}_{it}=a+b\ M{S_{EMA}}_{{}_{it}}+c\ {\left(M{S_{EMA}}_{{}_{it}}\right)}^2+c\ M{S_{ROW}}_{{}_{it}}+ Control\  variables+{\mu}_{it} $$

with *RD*_*it*_ as the logarithmic R&D expenditures divided by sales of firm *i* in time *t*. As robustness check, we also take the lagged values of the market share variables.

The explanatory variable *MSEMA* denotes the share of sales of firm *i* in Europe (including the markets of Middle East and Africa, which are still negligible in terms of size). As mentioned before, we also test for non-linear effects by including a squared term. The share of sales in the rest of the world (*MSROW*) is also included with Japan as the major representative of this group of countries. As the U.S. share of sales (*MSUSA*) is a residual of the sum of *MSEMA* and *MSROW* it is not necessary to explicitly add *MSUSA* into the equation. We include the following control variables:

- **Size:** The hypothesis that links firm size with R&D intensity goes back to Josef Schumpeter. In his early work he posited a negative relationship between firm size and R&D intensity arguing that small entrepreneurial firms are the engine of innovation [44]. In his later work however, he claimed that the major source of innovation were large corporations [45], which had also been observed in the early empirical literature [46]. For the pharmaceutical industry results are mixed: Some authors observed decreasing returns to R&D investments [47-49]. Others suggest significant returns to size in pharmaceutical research [50]. We take the logarithmic number of employees divided by sales (USD) to capture size effects (Employ).
- **Growth:** Growth rates of firms are also quite often linked to innovation. Hölzl [51] for instance found evidence that innovation activities and high-growth status are strongly dependent in Northern Europe. We simply take annual sales growth in percent as a control variable (Salesgr).
- **Leverage**: There is abundant empirical evidence that highly levered firms invest less in R&D [52-54] which might be due to capital market imperfections. We take logarithmic debt divided by sales to control for the corporate debt ratio (Leverage):
- **Tobin’s*q:*** Tobin’s *q* is the ratio of the market value of a firm to its assets and captures the investment opportunity differences across firms. Abstracting from financial market frictions, a firm invests up to the point where the marginal value of capital (marginal q) equals the marginal cost of capital. Under certain assumptions, the marginal value of capital equals the average value of capital (average q) [55], which we include into our equation (Tobin’s q).
- **Cash flow:** While a firm’s cash flow should not influence investments in perfectly functioning markets [56], the empirical literature tracing back to Meyer and Kuh [57] documents a strongly positive relationship between cash flow and investments. Either asymmetric information between investors and firms [58-60] or the separation of ownership and control, which leads to a principal-agent problem between a firm’s managers and its shareholders can be made responsible for this finding [61,62]. As most large pharmaceutical finance their investments firms with cash flow from existing products [63], we include the lagged logarithmic cash flow (USD) divided by sales (USD) (Cash Flow) into equation (1).

We believe that we include the most relevant determinants of R&D in our equation, although one might think of additional variables which influence R&D investments. For instance, on the firm level the impact of the degree of diversification [64], or organizational competence [65] have been studied. On a macro level, authors have taken a look at the role of public R&D spending and its spillover to corporate R&D [66,67].

We use panel data regression techniques, namely a fixed effects (FF) model and a random effects (RE) model. A fixed effects model might be more appropriate as the companies in our sample were not randomly chosen but based on firm size. The FE model also allows for correlation of unobserved heterogeneity with the explanatory variables [68]. On the other hand, the RE model is more efficient than the FE model when N is large, T is small and its assumptions are not violated. To pick the “correct” model is sometimes quite challenging and strongly dependent on the assumptions that have been made about the error component [69]. We therefore present results for both models.

## Results

Table 2 depicts the correlation matrix of the variables. It can be observed that a high share of European sales is positively correlated with company size and negatively correlated with the cash flow and investment opportunities as expressed by Tobin’s q. A high presence in the U.S. on the other hand goes along with a bigger cash flow, better investment opportunities, and a higher leverage ratio. A high R&D intensity is positively correlated to the U.S market share and inversely linked to the market share in EMA and ROW. Size seems also be negatively related to the R&D intensity.

**Table 2:** **Correlation matrix**

	**R&D**	**MS**_**EMA**_	**MS**_**ROW**_	**MS**_**USA**_	**Employ**	**Salesgr**	**Leverage**	**Tobin’s** ***q***	**Cash flow**
R&D	1								
MS_EMA_	−0.339	1							
MS_ROW_	−0.441	0.006	1						
MS_USA_	0.590	−0.683	−0.734	1					
Employ	−0.502	0.618	0.086	−0.482	1				
Salesgr	0.119	0.074	0.026	−0.069	−0.024	1			
Leverage	−0.180	0.004	−0.306	0.220	0.209	−0.128	1		
Tobin’s q	0.177	−0.203	−0.315	0.368	0.221	−0,021	−0.147	1	
Cash flow	0.138	−0.135	−0.095	0.161	−0.082	0.168	−0.133	0.332	1

The results of the multivariate regression are displayed in Table 3. The overall model fit is reasonably good although the difference between the within and overall R squared indicate the importance of individual firm effects [69].

**Table 3:** **Regression results**

	**Fixed effects**			**Random effects**		
	**Coef.**	**t**	**p > t**	**Coef.**	**t**	**p > t**
MS_EMA_	3.850	1.92	0.058*	2.118	1.34	0.179
(MS_EMA_)^2^	−5.704	2.23	0.023**	−3.946	1.84	0.066*
MS_ROW_	0.079	0.10	0.919	−0.867	2.42	0.015**
Employ	−0.106	1.83	0.071*	−0.111	1.93	0.053*
Salesgr	0.115	1.72	0.089*	0.098	1.50	0.134
Leverage	−0.063	3.88	0.000***	−0.058	4.14	0.000***
Tobin’s q	−0.016	1.15	0.252	−0.019	1.46	0.145
Cash flow	0.024	0.72	0.471	0.006	0.28	0.780
Constant	−3.287	−5.74	0.000***	−2.789	5.73	0.000***
R^2^	30.7% (within); 12.5% (overall)			30.0% (within); 52.0% (overall)		
Prob > p	0.000					
Prob > chi				0.000		

Significant at 10% level (*), 5% level (**), and 1% level (***).

The most important finding is a weak nonlinear relationship between *MS*_*EMA*_ and the R&D intensity. The higher the share of sales made in the EMA region, the higher is the negative impact of the squared term, in other words the more sales a company makes in the EMA region beyond a certain threshold the higher is the decline in R&D investment. This threshold value can be analytically derived by maximizing equation (1), i.e.

2 $$ \delta RD/{\delta MS}_{EMA}=b+2c\left(M{S}_{EMA}\right) = 0 $$

Rearranging leads to

3 $$ M{S}_{EMA}=-b/2c $$

Plugging the value of the coefficients *b* (3.85) and *c* (−5.70) into the equation yields 0.33. Hence, the maximal R&D intensity for a company is reached at 33 percent of its sales being generated in Europe. Above this threshold, a bigger presence in Europe seems to go along with decreasing R&D investments. However, this relationship is statistically significant only in the fixed effects model. To establish a stronger statistical link one would have to increase the number of observations for example by prolonging the sample period. The results do not alter much when the lagged values of the variables are used.

The control variables have the expected sign. The debt ratio is related to a lower R&D intensity while high growth firms seem to invest more in R&D. Size is negatively related to the R&D intensity as well. As both variables are expressed in logarithm we can interpret the coefficients as elasticity. Increasing employment by one percent is associated with a 0.11 percent decrease of R&D intensity.

## Discussion

We find that R&D intensity of a pharmaceutical company positively correlates to the sales the company makes in the U.S market, or to put it the other way round, it is inversely correlated to the fraction of sales generated in Europe. This result is a confirmation of Vernon’s [6] study with a more recent dataset. In contrast to Vernon whose left hand side variable was the corporate profit margin, we directly measure the effect on R&D spending. As the European market share serves as a proxy of pharmaceutical regulation in our analysis, we interpret the results as further evidence of the deteriorating effect of regulation on firm’s incentives to invest in R&D that has already been studies by Henry Grabowski [70]. Price regulation of pharmaceuticals can theoretically contribute to society’s total welfare by lowering drug prices and increasing health care access which results in higher consumer surplus. Conversely, the costs of regulation might offset this effect when the long term effects of reduced R&D spending are taken into account. While modeling welfare effects is beyond the scope of this paper, we are able to provide additional evidence for the detrimental effects of price regulation on pharmaceutical R&D spending. Another unintended effect of price regulation is that bio-pharmaceutical foreign direct investment is channeled into countries with less strict price controls [71]. Some European firms such as Novartis moved their entire R&D headquarters to the U.S. [72]. As a result, most new drugs are nowadays originated in the United States [73-76]. The dominance of the U.S. market also means that new drug development programs focus on disease areas with a high prevalence within the U.S. population such as obesity. Cholesterol lowering agents such as Pfizer’s Lipitor were the most successful drugs in the last decade. Because pharmaceutical research is primarily driven by profit expectations, high prevalence diseases of poorer countries are generally not in the focus of pharmaceutical R&D investment decisions [77]. On the other hand, some authors such as Light and Lexchin [78] claim that R&D investments do not necessarily lead to drug innovations. Instead, most R&D investments are channeled into low risk R&D programs that provide only minor clinical advantages over existing treatments. Future research should therefore shed more light on the quality aspects of the outcomes as regulation may not only decrease R&D spending but lead to a more efficient use. This argument has received some attention in the context of environmental regulation and is known as the “Porter Hypothesis” [79,80]. It was argued that a well-designed regulation can actually enhance competitiveness because it can trigger innovation. Applying this argument to the pharmaceutical industry regulation could in principle reduce the development of so called “me-too drugs” (i.e. drugs that are structurally very similar to already known drugs with only minor benefits to the patients) while maintaining or even increasing the number of break through innovations.

The rapid ageing of most European societies makes an easening of price controls and regulation in Europe rather unlikely in the near future. Pharmaceutical firms are probably forced to accept this challenge and need to respond with an improvement of R&D productivity [81]. Drawing on a data base with 28,000 compounds Pamolli et al. [82] observe a significant decline of R&D productivity since 1990 as the risk of failure is increasing. The trend of falling R&D productivity in the pharmaceutical industry has been termed ‘Eroom’s Law’ [83] in contrast to ‘Moore’s law’ that describes the productivity jumps in the semiconductor industry (in fact it is ‘Moore’s Law’ backwards). If the industry fails to increase productivity significantly, some industry observers foresee a gloomy future. As Paul et al. [84]: p214 put it: “Without a substantial increase in R&D productivity, the pharmaceutical industry’s survival (let alone its continued growth prospects), at least in its current form, is in great jeopardy.” In their model, a cost reduction of 50% per new chemical entity (NCE) is needed to sustain a viable R&D business model.” How the industry can meet this ambitious target remains an open issue that deserves further attention. Our results indicate that this problem is unlikely to be solved through mergers and acquisitions since company size is negatively related to R&D intensity. Outsourcing manufacturing and research and development tasks might another attempt to raise productivity [85,86]. This move on the other hand might lower the barriers for firms to enter the industry in the long run leading to even bigger pressure in the future [87].

Another trait of pharmaceutical regulation is that it does not only influence R&D investment decisions, but also determines the availability of new drugs. Studying the effect of external reference pricing Danzon et al. [88] found the launch delay of new drugs to be positively related to expected price. Kyle’s [89] research confirmed those findings. In addition, she concluded that drugs invented by firms headquartered in countries that use price controls reach fewer markets and with longer delays than products that originates in countries without price controls. Another effect of regulation is a change of the drug market structure. Older work that has studied the effects of the FDA regulation in the U.S. following the Thalidomide drug scandal find smaller firms to be more affected than larger firms [90,91]. Many small firms that did not have access to financial resources were not able to cope with the tighter regulation and had to leave the market resulting in an increase of market concentration.

## Conclusions

Summing up, the high R&D intensity of the pharmaceutical industry largely depends on the unregulated and profitable U.S. market, making many Americans complain about European “free riding” on U.S. R&D expenditures that are primarily borne by American patients [92,93]. As the mere amount of R&D expenditures is only an input factor within the innovation process future research should take a closer look at the impact of regulation on the efficiency of R&D expenditures. An increase of R&D investment makes economically only sense if it leads to more drugs that meet an unmet medical need and make a significant difference to the patient.
